# Sonic horizon formation for oscillating Bose-Einstein condensates in isotropic harmonic potential

**DOI:** 10.1038/srep38512

**Published:** 2016-12-06

**Authors:** Ying Wang, Yu Zhou, Shuyu Zhou

**Affiliations:** 1 School of Mathematics and Physics, Jiangsu University of Science and Technology, Zhenjiang 212003, China; 2 Key Laboratory for Quantum Optics, Shanghai Institute of Optics and Fine Mechanics, The Chinese Academy of Sciences, Shanghai 201800, China

## Abstract

We study the sonic horizon phenomena of the oscillating Bose-Einstein condensates in isotropic harmonic potential. Based on the Gross-Pitaevskii equation model and variational method, we derive the original analytical formula for the criteria and lifetime of the formation of the sonic horizon, demonstrating pictorially the interaction parameter dependence for the occur- rence of the sonic horizon and damping effect of the system distribution width. Our analytical results corroborate quantitatively the particular features of the sonic horizon reported in previous numerical study.

Since the first experimental realization of Bose-Einstein condensation (BEC) in 1995, there is a lot of experimental and theoretical work focusing on the dynamical properties of the ultracold system. The nonlinear phenomena, like soliton, vortex formation and evolution in the BEC system are the hot topics within the past decade in BEC related studies^1^,^2^,^3^,^4^,^5^,^6^,^7^. The particular nonlinear features, the categorization of bright/dark soliton for example, are dependent on the nonlinear inter-particle interaction constant in the theoretical model, for which the Gross-Pitaevskii equation (GPE) is chosen and proved to be pretty reliable. It is now known that the amplitude and sign of scattering length (*a*_*s*_) determine the strength and the sign (attractive or repulsive) of the nonlinear inter-particle interaction. It is now possible to tune the amplitude and sign of the scattering length through magnetically controlled Feshbach resonance technique, so that the long pursued Bardeen-Cooper-Schrieffer to BEC crossover is realized experimentally^8^,^9^.

Given the flexible tunability, besides typical nonlinear features, ultracold system is the ideal choice for investigating many intriguing physics, among these is the ultracold physics analog of black hole event horizon in the exploration of cosmology and gravitational physics. This corresponds to the use of artificial black holes^10^,^11^. As indicated by Unruh in his seminal paper^12^, the supersonic flow excitations corresponds to a scalar field equation on a curved spacetime containing a horizon. The corresponding ultracold fluid analog is sonic horizon, when identifying the fluid flow with curved spacetime and excitation mode with curved spacetime fields. Due to their ultracold temperature and well isolation, trapped Bose-Einstein condensates were proposed as promising candidates for observing sonic black holes and Hawking radiation^13^. Experimental demonstration of sonic black holes^14^ and Hawking radiation^15^ had been realized by accelerating an elongated condensate in a step like potential. It is shown by numerical study^16^ that for a static ground state BEC system trapped in isotropic harmonic potential, when there is abrupt change of scattering length via Feshbach resonance technique, the system will expand/contract with time and under certain parametric setting there exists sonic horizon which is the spherical surface outside which the fluid flow speed surpass that of sound. Here in this paper we will perform an analytical study of the evolution of similar initial BEC system investigated in prior numerical study^16^. We utilized the Gross-Pitaevskii equation (GPE) model, through the modified variational approach, calculated the criterion for the formation and lifetime of the sonic horizon, and quantitatively corroborate the sonic horizon feature shown in prior work^16^. Also the damping effect arising from phonon excitation is analyzed numerically with pictorial demonstration.

This paper is arranged as follows, the next section makes the theoretical model analysis and gives the detailed calculation steps combined with pictorial demonstration regarding the key collective features of the system. The conclusive remarks are made in the last section.

## Methods

### The GPE model and modified variational ansatz

The study of the formation of sonic horizon could be carried out in expanding BEC in isotropic harmonic trap 
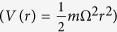
, in which an abrupt change in nonlinear interaction strength is the cause of the expansion. As in prior work, the Gross-Pitaevskii equation could be chosen as the theoretical model, but in order to give more precise description of the nonlinear interaction, tunable interaction strength parameter is incorporated in the model. The corresponding equation takes the following format,


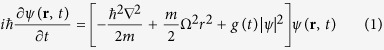


The systems described by Eq. (1) start to evolve from the state *ψ*_0_ = *ψ(***r**, 0) which solves the stationary equation of Eq. (1) with *g(t* ≤ 0) = *g*_0_. During the evolution, *g(t*) = *g (t* > 0). *g(t*) depends on the inter-particle scattering length which is adjusted through the Feshbach resonance technique. Assume initially (*t* < 0), the BEC system is in the ground state with distribution width *σ*_0_. The initial wave function (*t* = *t*_0_) takes the following form,


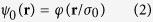


where *φ(r*) vs. *r* functional curve is very similar as that of exp (−*r*^2^/2) (zero nodes, decay from maximum value at *r* = 0 to minimum value 0 at +∞), its actual form is discussed in the ensuing steps. Different from regular variational treatment, here we choose the parameterized functional form *φ* instead of regular gaussian function to reach more precise description of the system. While it is generally true that *φ* may evolve into inexplicit form that is difficult to determine analytically, the evolution of the distribution width may bear significant physical meaning and can be solved analytically. Consider the following action of Eq. (1) 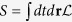 with the Lagrangian density expressed as,


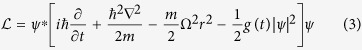


We use the following ansatz for *ψ*(**r**, *t*) (for d-dimensional case),


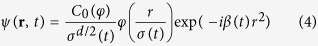


There are three real single-variable functions *φ(x*) (*x* = *r/σ(t*)), *σ(t*) and *β(t*) to be determined, *C*_0_(*φ)* is normalization constant determined by function *φ(x*) according to,


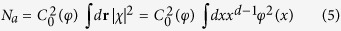


where *N*_*a*_ is the total number of constituent particles.

Based on the formulation (3) and ansatz (4), we show in the subsection the principle calculation steps towards the typical collective mode of the system.

### Oscillation mode

Substitute ansatz (4) into Eq. (1) and set the imaginary part to zero, we can directly obtain,


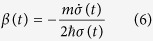


Combine with (6), the variation of action *S* with respect to *σ* and *φ* gives two coupled equations for *φ(x*) and *σ(t*) as follow,


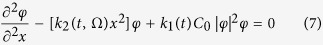



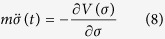


where 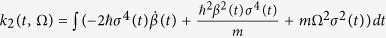, *k*_1_(*t*) = ∫*g*_1_(*t)σ*^2^(*t)dt*, and *V(σ*) = *V*_0_(*σ*) + *V*_1_(*σ*), with


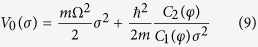



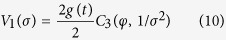


*C*_1_(*φ*), *C*_2_(*φ*) depend on *φ, C*_3_(*φ*) depends on *φ* as,


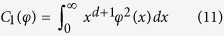



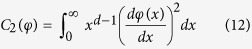



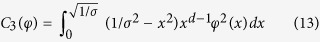


For weak nonlinear interaction (*g(t*) very small) which warrant neglecting *V*_1_(*σ*), Eq. (8) is integrable from (10) and we can solve for *σ(t*) as follow,


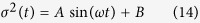


where


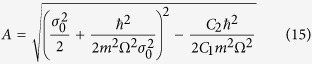



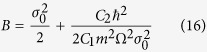



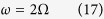


*σ*_0_ is the initial distribution width at *t* ≤ 0, with 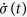 reflecting the system’s expansion/contraction speed for *t* > 0. We can see that *σ(t*) oscillates with period 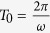. We can also see that the distribution width *σ(t*) oscillates between the maximum value 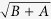 and minimum value 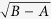 and is just the breathing mode arising from the quantum pressure.

## Results and extended analysis

### Criterion and lifetime for the formation of the sonic horizon

Based on the analytical results regarding the oscillation mode derived in the previous section. We calculate the key physical quantities bearing significant physical meaning.

From formula (6), we can get the fluid velocity as,


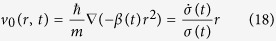


The sound velocity^17^ in the weak interaction limit is


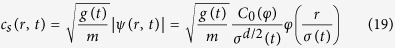


where *φ(x*) possesses the same qualitative feature as 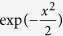 and is decreasing function of *r* for fixed time *t*, whereas *v(r, t*) is an increasing function of *r*. Suppose the BEC system is bounded as *r* ≤ *R*, the bounded function 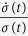 has maximum value *β*_*m*_ at *t* = *t*_*m*_ in the range 0 ≤ *t* ≤ *T*_0_, when *β*_*m*_*R* > *c*_*s*_(*R, t*) holds, there is timing range [*t*_1_, *t*_2_] with *t*_1_ ≤ *t*_*m*_ ≤ *t*_2_ such that *v*_0_(*r, t*) = *c*_*s*_(*r, t*) or


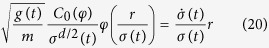


has solution *r*_*c*_ (0 < *r*_*c*_ ≤ *R*) and *r*_*c*_ is just the critical radius corresponding to the sonic horizon, below (above) which the fluid flow is subsonic (transonic).

It is not hard to see that the time *t*_*m*_ for the appearance of 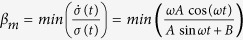 is,


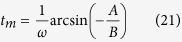


with


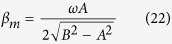


The lifetime of *τ* = *t*_2_ − *t*_1_ can be evaluated as follow. Around *t* = *t*_*m*_, both *v(r, t*) and *c*_*s*_(*r, t*) are decreasing function of *t*, so *t*_1_ = *t*_*m*_, but for significantly large *R, c*_*s*_ decreases much faster than *v(r, t*) and coincides approximately at zero value which corresponds to 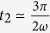 in reference to *v(r, t*) ∝ cos(*ωt)r*. So the lifetime of the sonic horizon is


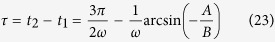


**Figure 1:**
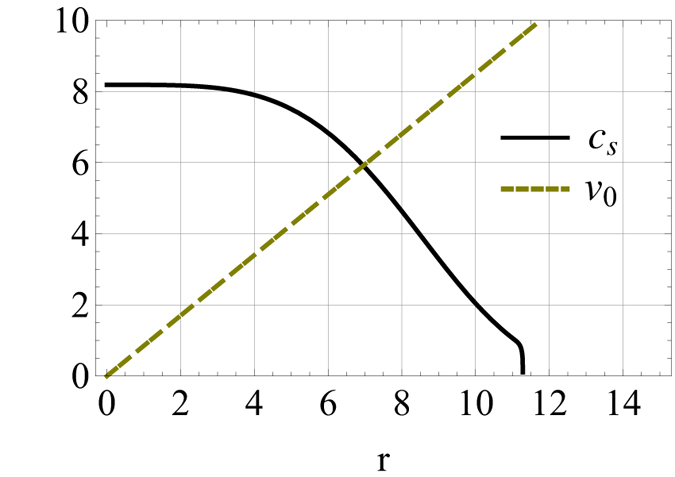
Sound velocity *c*_*s*_ (solid line, based on formula (19)) and fluid velocity *v*_0_ (dashed line, based on formula (18)) in units of (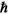
Ω/*m*)^1/2^ vs. *r* (in units of (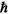/*m*Ω)^1/2^) at 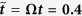, with *a*_*i*_ = 200*a*_0_, *a*_*f*_ = 5*a*_*i*_.

**Figure 2:**
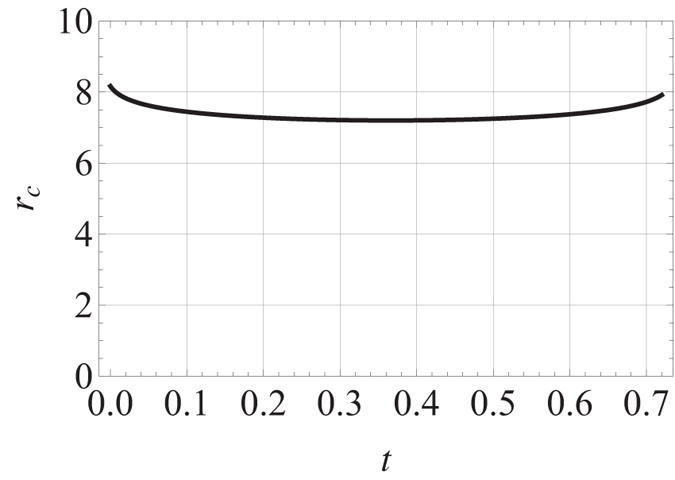
Position of sonic horizon *r*_*c*_ (based onEq. (20)) vs. 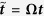 in units of (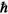/*m*Ω)^1/2^ for *a*_*i*_ = 200*a*_0_, *a*_*f*_ = 5*a*_*i*_.

**Figure 3:**
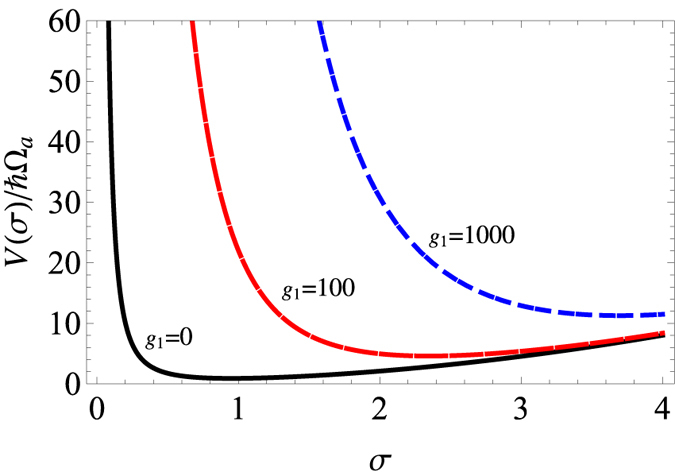
*V(σ*) vs. *σ* (in unit of> 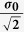) for three different nonlinear interaction constants: *g*_1_ = 0, 100, 1000 in unit of 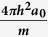, *a*_0_ is the initial s-wave scattering length.

Figure 1 shows the variation of *v*_0_, *c*_*s*_ with *r* for 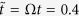 from Eqs (18) and (19), which cross at *r*_*c*_. We can see that the theoretically derived functional curves of *v*_0_ and *c*_*s*_ match very well with the results (shown as Fig. 2 in prior work^16^) that are obtained from numerical simulation. Figure 2 shows the variation of *r*_*c*_ (units of (*ħ/m*Ω)^1/2^) vs. *t* (units of Ω^−1^) with *r*_*c*_ obtained from solving algebraic equation (20). We can see that our analytical results shown by these two figures agree very well with that reported by prior pure numerical analysis (shown in Fig 3 in ref. 16 with same timing range). We can see that although in the timing range under study, the system’s distribution width varies significantly, but in the intermediate region of *r* around *r*_*c*_, *c*_*s*_ (∝|*φ*|^2^) varies in pace with *v*_0_ (∝*r*), so the crossing point *r*_*c*_ between curves of *c*_*s*_ and *v*_0_ is almost a constant in the timing range under study. Physically this means that the position of the sonic event horizon is relatively stable in most of the timing range under study. For the case of *g*_1_(*t*) ≠ 0, when the effect of *V*_1_(*σ*) has to be taken into account as the magnitude of *g*_1_(*t*) increase, *σ(t*) may not possess analytical solvable format as formula (14). But when the magnitude of *g*_1_(*t*) is not so big 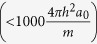, the *g*_1_(*t*) term could be treated as perturbation, we anticipate the system showing the same qualitative feature as the case with *g*_1_(*t*) = 0. This can be seen when we plot the ‘potential’ curve of *V(σ*) (incorporating *V*_1_(*σ*)) to investigate its quasi-static mode. From Fig. (3), we can see for *g(t*) = *g* (constant) that is not too big 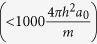, the quasi-static oscillation mode (around local minimum of *V(σ*)) exists and as shown by formula (23), the lifetime of sonic horizon is of order 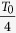, if it appears when *β*_*m*_*R* > *c*_*s*_(*R, t*) holds.

### Damped oscillation

The theoretical treatment made in the previous section does not consider the energy loss due to quantum fluctuation. Generally the energy loss due to the creation of Bogoliubov phonons will lead to the damping of the dynamical evolution. The Hamiltonian incorporating the Bogoliubov excitation reads^18^:


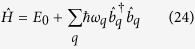


where *ψ*′ = *ψ* + *δψ, ψ*_0_ is the condensed part, 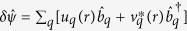, *u*_*q*_, *v*_*q*_ solve the Bogoliubov-de Gennes equations, with 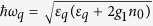 are the phonon energies, 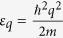 are the free particle energies^19^. The formulation of Lagrangian 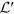 from 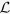 is as follows,


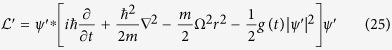


The spatial integral of 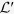 change from that of 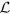 as follows,


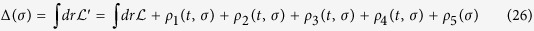


where


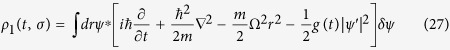



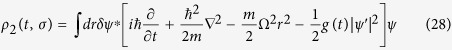



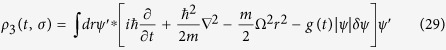



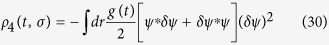



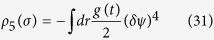


**Figure 4:**
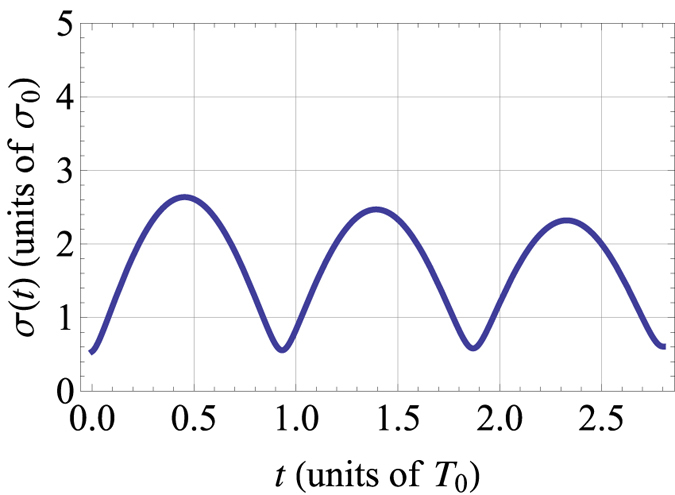
Damped oscillation of *σ(t*) with time *t* for *a*_*i*_ = 200*a*_0_, *a*_*f*_ = 5*a*_*i*_.

The expression for *V(σ*) (in Eq. (8)) will incorporate an additional term 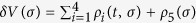 in addition to *V*_1_(*σ*) (Eq. (9)), which is equivalent to adding a damping term 
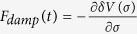
 on the right hand side of Eq. (7b) whose detailed effects can be shown by numerical simulation. The damped oscillation is shown in Fig. 4. But the appearance of *r*_*c*_ (sonic horizon) is still periodic although the oscillatory magnitude of the system’s distribution width gradually reduces due to the energy loss from phonon excitation.

## Conclusion

In this paper, based on the Gross-Pitaevskii equation and modified variational method, we calculate the evolution of Bose-Einstein condensates in isotropic harmonic potential when it suddenly experiences an abrupt change of s-wave scattering length via Feshbach resonance technique. We show through our analytical calculation that under certain condition, the fluid flow velocity can surpass that of sound beyond certain critical radius which signals the occurrence of sonic horizon. We derive the original analytical formula for the lifetime of the sonic horizon which agrees quantitatively with that reported in prior work with numerical simulation. The effect of quantum fluctuation is studied numerically and the damping phenomenon of the system distribution width *σ(t*) is identified. We also show pictorially the interaction strength dependence of the existence and stability trend of the quasi-static oscillation mode, demonstrating the applicability of the theoretical treatment presented in our work.

## Additional Information

**How to cite this article**: Wang, Y. *et al*. Sonic horizon formation for oscillating Bose-Einstein condensates in isotropic harmonic potential. *Sci. Rep.* **6**, 38512; doi: 10.1038/srep38512 (2016).

**Publisher's note:** Springer Nature remains neutral with regard to jurisdictional claims in published maps and institutional affiliations.
